# Ingestion of attractive toxic sugar baits containing ivermectin before and after blood feeding affects the biology and reproduction of *Aedes aegypti* (Diptera: Culicidae)

**DOI:** 10.1111/mve.70065

**Published:** 2026-03-29

**Authors:** Thais Alves de Moura, Alyne Cunha Alves Dias, Alexandre de Almeida e Silva

**Affiliations:** ^1^ Department of Biology Federal University of Rondonia Porto Velho Brazil; ^2^ National Institute of Epidemiology of Western Amazonia, Fiocruz‐Rondonia Porto Velho Brazil

**Keywords:** *Aedes aegypti*, ivermectin, reproduction, survival, vector control

## Abstract

The attractive toxic sugar bait (ATSB) is an alternative approach for mosquito control, combining an attractant, a phagostimulant (sugar) and an insecticide. In this study, we evaluated the effects of ATSBs containing ivermectin (IVM, 15 ppm) on the biology and reproduction of *Aedes aegypti* females under different conditions relative to blood feeding. The bait was prepared with guava juice, 70% brown sugar, water, food dye and IVM. Females aged 5–7 days from the LaBEIn colony were allocated to two treatment groups: bait before blood feeding (BB) and bait after blood feeding (AB). Each group consisted of 20 females, with three replicates, and the treatments were applied 48, 72, or 96 h BB or AB. Blood engorgement was reduced by approximately 80% in all BB treatments during the first gonotrophic cycle, while no effect was observed in AB females. Survival decreased by up to 90% in BB females at all time points, whereas AB females exhibited a 94% reduction only at 96 h. For reproductive traits, including oviposition rate, egg number and hatching, the strongest reductions occurred in BB females (up to 80%) and in AB females exposed 96 h AB, also during the first gonotrophic cycle. Overall, ATSB‐IVM ingestion caused pronounced sublethal effects on *Ae. aegypti*, impairing blood feeding, survival, oviposition, fecundity and fertility, with greater impacts when ingestion occurred BB and during the first gonotrophic cycle. These results highlighted the potential of ATSB‐IVM as a complementary strategy for vector control.

## Graphical abstract

- Attractive toxic sugar bait containing ivermectin (ATSB‐IVM) ingestion markedly reduced blood feeding, survival, oviposition, egg production and larval hatching, with the strongest effects when baits were ingested before blood feeding.
- Timing of ingestion (48–96 h before or after blood feeding) shaped sublethal impacts across the first and second gonotrophic cycles, altering key reproductive parameters.
- Low‐dose ATSB–IVM produced lasting sublethal effects that impaired mosquito reproductive fitness, indicating its potential as a complementary vector control strategy.

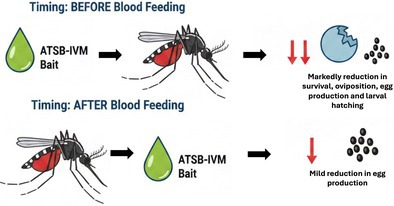

## INTRODUCTION

The rapid spread of arboviruses such as dengue, Zika, chikungunya and yellow fever, transmitted by *Aedes* *aegypti* (Linnaeus, 1762), has led to recurrent epidemics in Brazil in recent years (Medeiros, 2024; Valente et al., 2024; Mendonça et al., 2023). These public health challenges underscore the urgent need for innovative and sustainable strategies to achieve effective vector control (Javed et al., 2022).

One promising approach is the attractive toxic sugar bait (ATSB), designed to lure and kill mosquitoes through the ingestion of toxic compounds combined with a phagostimulant, typically sucrose. ATSBs have been proposed as complementary tools for the control of *Anopheles* Meigen, 1818 and *Aedes* Meigen, 1818 mosquitoes and have demonstrated effectiveness in both outdoor (Müller et al., 2008, 2010) and indoor field trials (Qualls et al., 2015).

ATSB formulations may incorporate natural and/or synthetic toxicants, including garlic oil (Junnila et al., 2015), boric acid (Barbosa et al., 2019) and various insecticide classes such as pyrethroids, phenylpyroles, pyrroles, neonicotinoids and macrocyclic lactones (Allan, 2011). Among these, ivermectin (IVM), a well‐known endectocide, has shown particularly high efficacy, with reported mortality rates of 93% in *Anopheles* *stephensi* Liston, 1901, 99% in *Anopheles culicifacies* Giles, 1901 (Kumar et al., 2023), 95% in *Anopheles arabiensis* Patton, 1905 (Tenywa et al., 2017) and over 90% in *Ae. aegypti* (Dias et al., 2023, 2025; Tenywa et al., 2022).

In addition to its mosquitocidal effect, IVM has been shown to impair mosquito reproduction. When ingested during blood feeding, it reduces fecundity and fertility in *Anopheles aquasalis* (Sampaio et al., 2016), *Anopheles coluzzii* Coetzee & Wilkerson, 2013 (Pooda et al., 2024), *An. arabiensis* (Mekuriaw et al., 2019) and *Ae. aegypti* (Mahmood et al., 1991).

However, no study to date has addressed the sublethal impacts of ATSB‐IVM on the reproductive potential of *Ae. aegypti*, effects that have already been documented when IVM is acquired through host blood. To fill this gap, the present study evaluated the effects of ATSB‐IVM ingestion on the fecundity and fertility of *Ae. aegypti* when consumed either before blood feeding (BB) or after blood feeding (AB) across different gonotrophic cycles under controlled laboratory conditions.

## MATERIALS AND METHODS

### Ethical aspects

All procedures for mosquito rearing and blood collection for feeding were approved by the Research Ethics Committee of the Federal University of Rondônia under protocol number CAAE: 78543817.9.0000.5300.

### Laboratory rearing of Ae. aegypti

For the experiments, *Ae. aegypti* females from the Insect Bioecology Laboratory (LaBEIn) colony were used, following the rearing protocol described by Barbosa et al. (2019). Briefly, the eggs were transferred to containers (30 × 22 × 6 cm) with approximately 250 mL of mineral water and fed with Reptile Feed (Reptolife) until pupation. The adults were maintained in screened cages and provided with a 10% sucrose solution. Three days post‐eclosion, females were separated from males and subjected to a 48‐h fasting period before being fed with either blood or sugar bait for the assays. Mosquitoes were maintained under controlled insectary conditions at 28°C and 70–80% relative humidity.

### Preparation of attractive sugar bait and ATSB with IVM

The attractive sugar bait (ASB), used as the control, was prepared with concentrated guava juice (Maguary®) and filtered water (3:1), 70% brown sugar and 3% green food dye (Arcólor®). The solution was left to stand for 48 h before use. To prepare the ATSB‐IVM, the ASB solution was supplemented with IVM at a concentration of 15 ppm, using the veterinary formulation Ivomec® (1% IVM for cattle).

### Evaluation of ATSB ingestion BB and AB

The experiments were designed to assess the effects of ATSB ingestion either BB or AB at three time intervals: 48, 72 and 96 h. Assays were conducted in screened cages (500‐mL plastic cups).

To evaluate the effect of ATSB or ASB (control) ingestion prior to blood feeding (BB), ATSB and ASB were offered for 6 h on cotton pads to 360 females starved for 48 h. After exposure, the cotton pads were removed, and mosquitoes were anaesthetised to assess engorgement levels under a stereomicroscope. Only females with an engorgement grade ≥2 were selected, while those with lower grades were discarded (Pilitt & Jones, 1972). A subset of 20 engorged females was returned to their cages, provided with 10% sucrose for 24 h and then starved for 24 h BB for the first experimental group (48 h after bait ingestion). A second subset of 20 females was maintained in cages, fed ad libitum with sucrose for 48 h and then starved for 24 h BB for the second experimental group (72 h after bait ingestion). A third subset of 20 females was kept in cages, fed with sucrose for 72 h and subsequently starved for 24 h BB for the third experimental group (96 h after bait ingestion).

Blood meals were offered using 50‐mL cups containing human blood at the bottom, sealed with Teflon tape to create an artificial membrane and positioned laterally to allow mosquito feeding (Siria et al., 2018). Two days AB, blood‐fed females were individually transferred to oviposition cages consisting of 180‐mL plastic cups lined with filter paper and covered with mesh for 48 h. To moisten the oviposition substrate, 20 mL of distilled water was added.

To evaluate the effect of blood feeding prior to ATSB or ASB (control) ingestion (AB), human blood was offered as described above to 360 females. A subset of 20 engorged females was returned to their cages and starved for 48 h before receiving ATSB or ASB (control) for the first experimental group (48 h AB). A second subset of 20 females was kept in cages, fed with sucrose for 24 h and then starved for 48 h before receiving ATSB or ASB (control) for the second experimental group (72 h AB). A third subset of 20 females was kept in cages, fed with sucrose for 48 h and then starved for 48 h before receiving ATSB or ASB (control) for the third experimental group (96 h AB). After bait feeding, mosquitoes were anaesthetised and assessed for engorgement grade under a stereomicroscope. Only females with an engorgement grade ≥2 were selected and individually transferred to oviposition cages as previously described.

All experiments were repeated across three different mosquito generations. Within each generation, experiments were performed using three independent groups of mosquitoes (biological replicates, *N* = 3), each composed of distinct individuals sampled from the colony. Thus, a total of nine independent biological units were evaluated in the study.

### Biological parameters measured

The number of blood‐fed females was used to calculate blood‐feeding rate (engorged females ÷ initial females). Survival rate was calculated as the number of females up to oviposition ÷ bait/blood engorged females. Oviposition rate was estimated as the number of females that laid eggs ÷ surviving females AB. Fecundity was determined by counting the number of eggs per female under a stereomicroscope. The filter papers containing eggs were dried for 48 h and then submerged in plastic trays with distilled water and eclosion rate was estimated after 5 days, counting the number of larvae ÷ number of eggs laid.

After oviposition in the first gonotrophic cycle, surviving females from all treatments were transferred to new individual cages and simultaneously offered a second blood meal to evaluate residual effects of ATSB ingestion (BB or AB) on the second gonotrophic cycle.

### Statistical analysis

Survival rate, bait engorgement, blood feeding, egg number and hatching rate were analysed using two‐way ANOVA (factors: bait composition, i.e., ATSB‐ASB and time, i.e., 48, 72, 96 h). To ensure independence across replicates, each of the nine biological units evaluated consisted of distinct individuals sampled from the colony across three different mosquito generations. This approach was specifically chosen to capture the inherent biological variability of the colony while maintaining the independence of observations. Pairwise comparisons among groups were performed with Sidak's test using GraphPad Prism 10 (GraphPad Inc.).

## RESULTS

**FIGURE 1:**
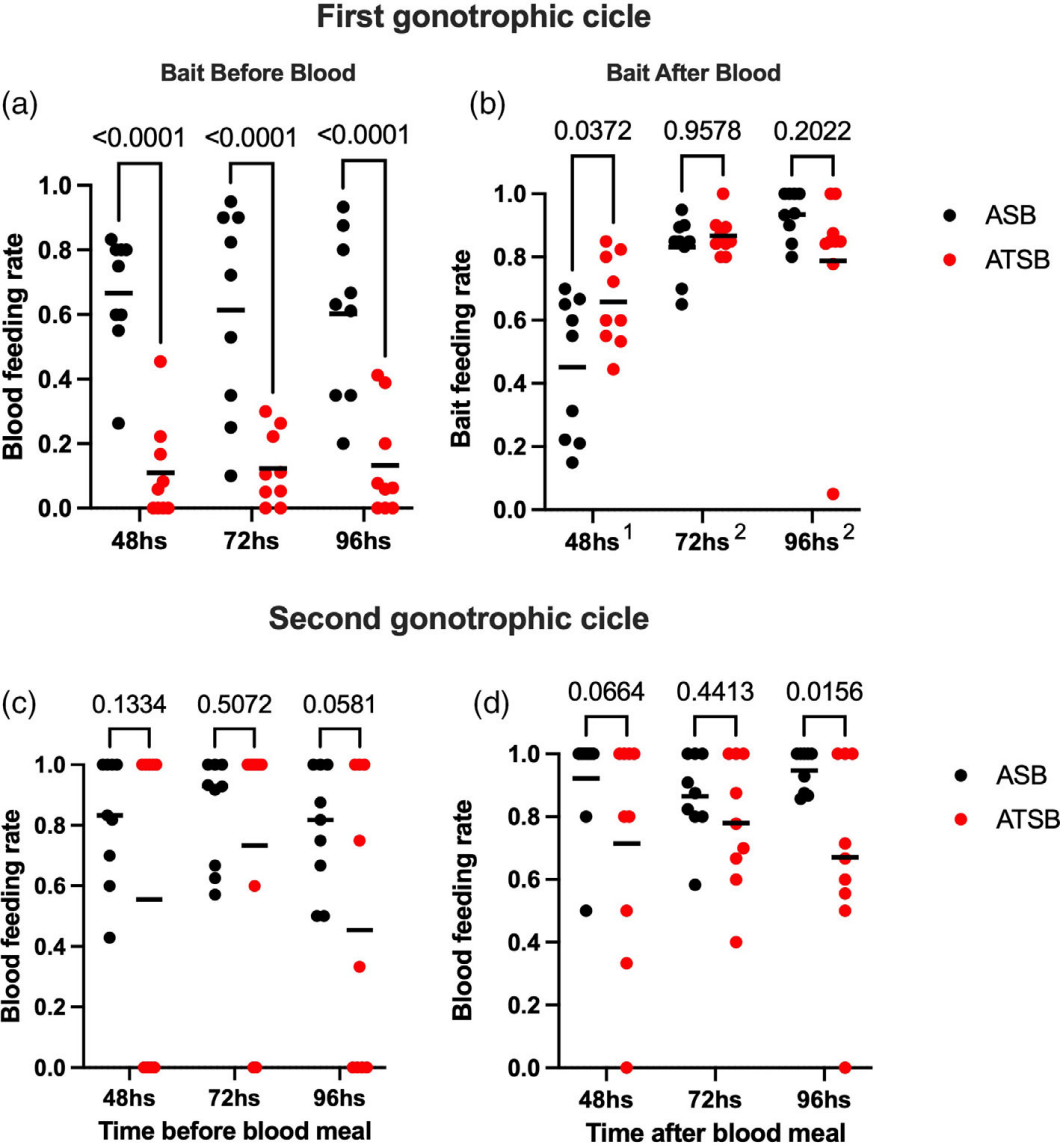
Blood‐ or sugar‐bait engorgement rates of *Aedes aegypti* females exposed to attractive toxic sugar baits (ATSBs) before (a) and after (b) blood feeding during the first gonotrophic cycle and blood‐feeding rates only during the second gonotrophic cycle (c, d) to evaluate residual effects of previous treatments. Different numbers indicate significant differences (*p* < 0.05) among time intervals in (b). Numbers over attractive sugar bait (ASB) and ATSB within a given interval indicate *p* values.

ATSB ingestion BB reduced the blood‐feeding rate by approximately 80%, regardless of the time interval (24 h, 48 h or 72 h) (*F* = 78.9; *p* < 0.0001) (Figure 1a). In contrast, in the group that received ATSB AB, there was no reduction in sugar‐bait engorgement by females (*F* = 0.49; *p* = 0.48). However, we observed a significant increase in bait engorgement 72 h after the blood meal (*F* = 18.9; *p* < 0.0001), reaching 0.86 after 96 h (Figure 1b).

During the second gonotrophic cycle, an overall reduction in the blood feeding was observed when mosquitoes had previously fed on ATSB in the previous gonotrophic cycle BB (*F* = 5.7; *p* = 0.03) or AB (*F* = 5.7; *p* = 0.03). Despite that, no significant changes were detected in blood‐feeding rates among surviving females that had ingested ATSB BB in pairs for each time compared (*F* = 2.91; *p* = 0.10) (Figure 1c). In the AB group, however, a reduction in blood‐feeding rate was observed only among females that ingested ATSB 96 h AB (0.67), compared with those that received ASB (0.94) (Figure 1d).

Overall, we observed reduced survival in groups that received ATSB compared with ASB (control) during the first gonotrophic cycle, regardless of the timing of bait exposure, that is, BB (*F* = 51.97; *p* < 0.0001) and AB (*F* = 6.59; *p* = 0.013). A similar reduction was also detected during the second gonotrophic cycle in both BB (*F* = 19.36; *p* < 0.0001) and AB groups (*F* = 7.04; *p* = 0.010).

**FIGURE 2:**
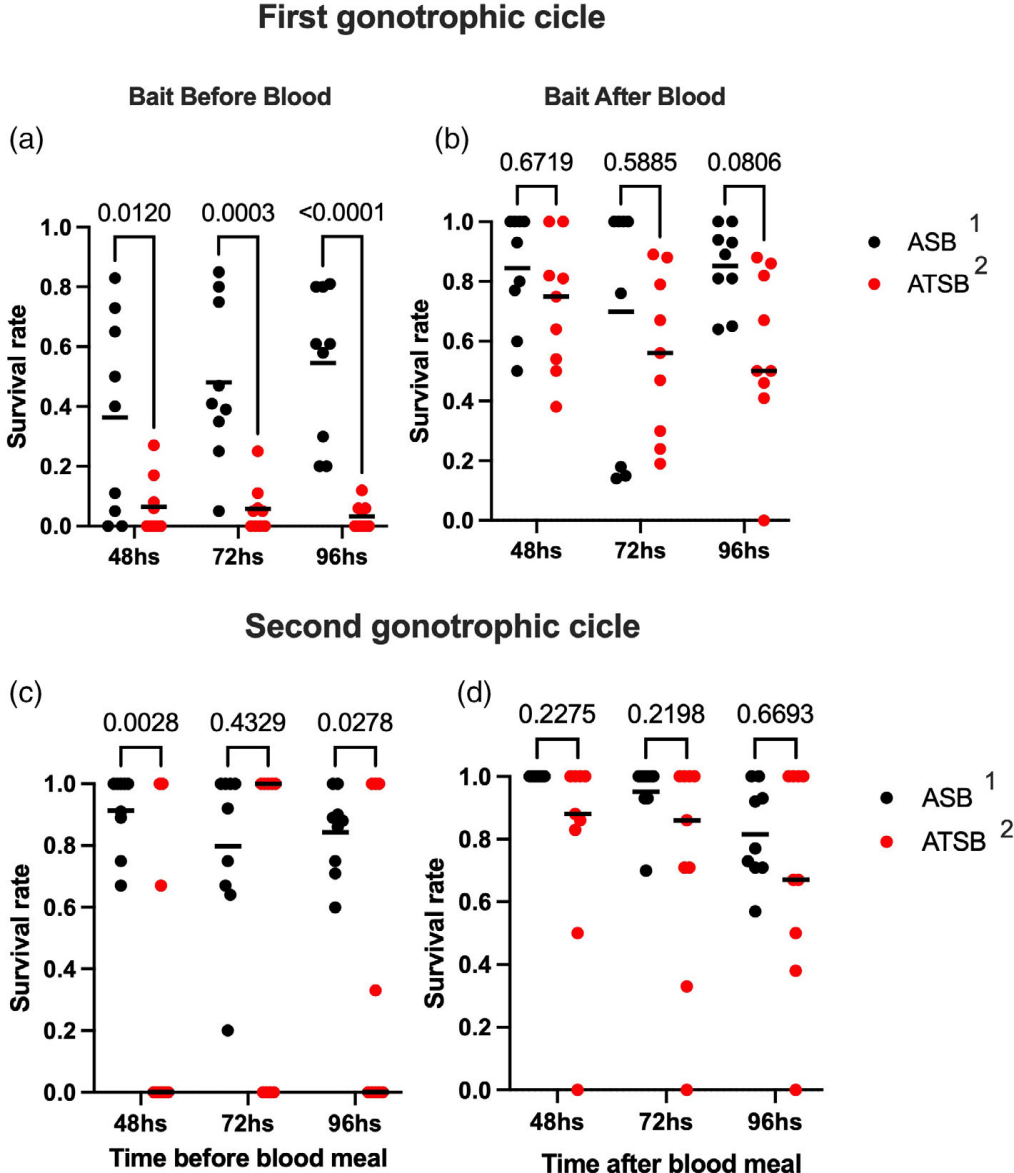
Survival rates of *Aedes aegypti* females exposed to attractive toxic sugar baits (ATSBs) at different time points, that is, before (BB) and after (AB) blood feeding during the first gonotrophic cycle and only blood feeding during the second gonotrophic cycle to evaluate the residual effect of previous treatments. Different numbers indicate significant differences (*p* < 0.05) between attractive sugar bait (ASB) and ATSB. Numbers over ASB and ATSB within a given interval indicate *p* values.

Survival of females that ingested ATSB BB decreased by up to 94% when the blood meal was provided before blood for all times evaluated (Figure 2a). However, no significant reduction (*p* > 0.05) was observed between ASB and ATSB‐fed females in the AB group (Figure 2b). A similar pattern was also observed in the BB groups in the second gonotrophic cycle, except for 72 h (Figure 2c), but not in the AB groups (Figure 2d).

**FIGURE 3:**
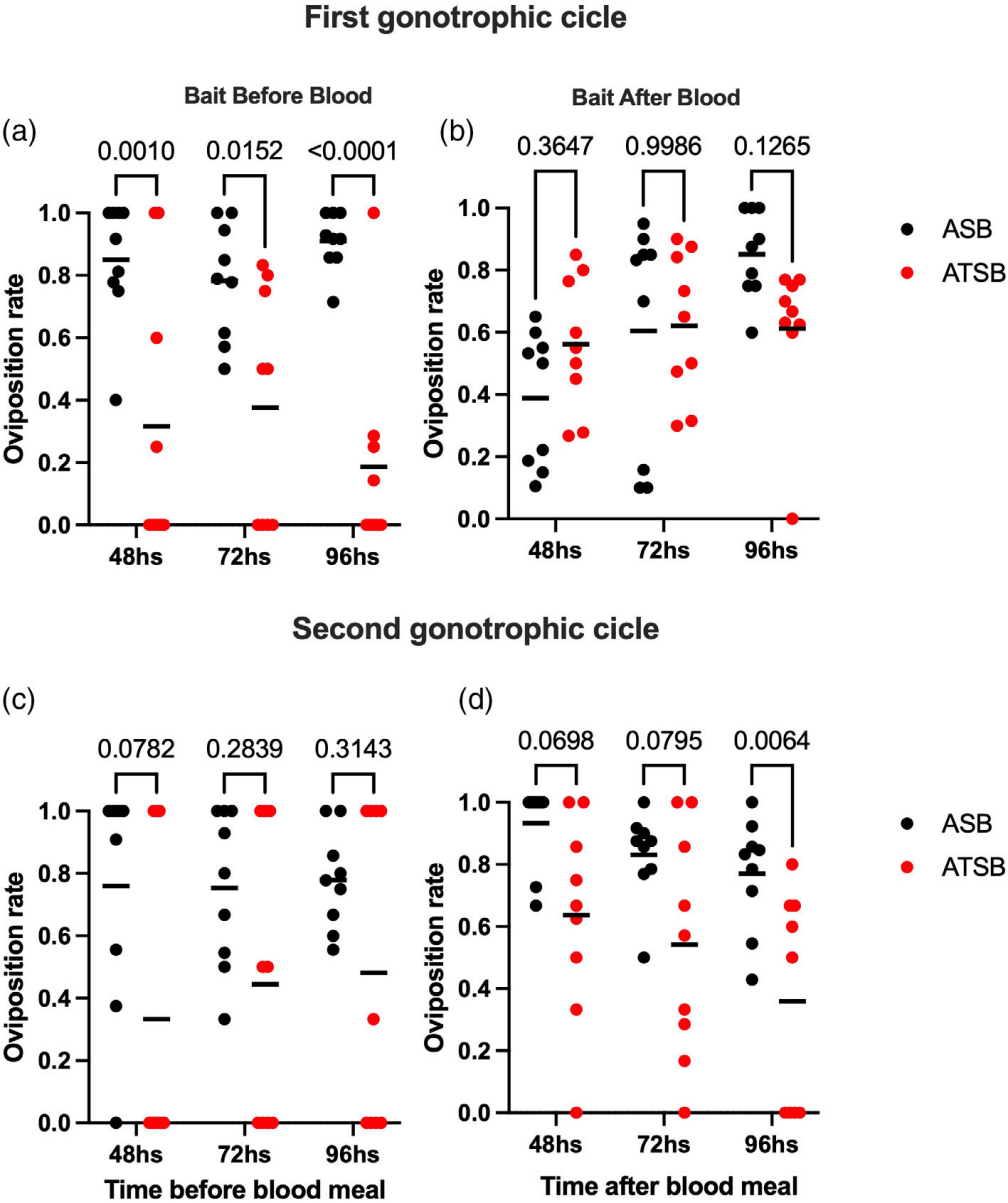
Oviposition rate of *Aedes aegypti* females exposed to attractive toxic sugar baits (ATSBs) at different time points, that is, before (BB) and after (AB) blood feeding during the first cycle and only blood feeding during the second gonotrophic cycle to evaluate residual effects of previous treatments. Numbers over attractive sugar bait (ASB) and ATSB within a given interval indicate *p* values.

During the first gonotrophic cycle, the oviposition rate of females that received ATSB BB was reduced regardless of the time at which the bait was offered (*F* = 48.02; *p* < 0.0001), with the highest reduction (80%) observed when bait was provided 96 h BB (Figure 3a). In contrast, there was no change in oviposition rate when females received ATSB AB compared with those that received ASB (control) (*F* = 0.06; *p* = 0.80) (Figure 3b).

In the second gonotrophic cycle, the oviposition rate of groups that had previously received ATSB either BB (*F* = 10.18; *p* = 0.0025) (Figure 3c) or AB (*F* = 20.6; *p* < 0.0001) (Figure 3d) was lower (30%) compared with ASB‐fed controls during the first cycle. However, significant pairwise differences were observed only for females that received ATSB 96 h AB (Figure 3d).

**FIGURE 4:**
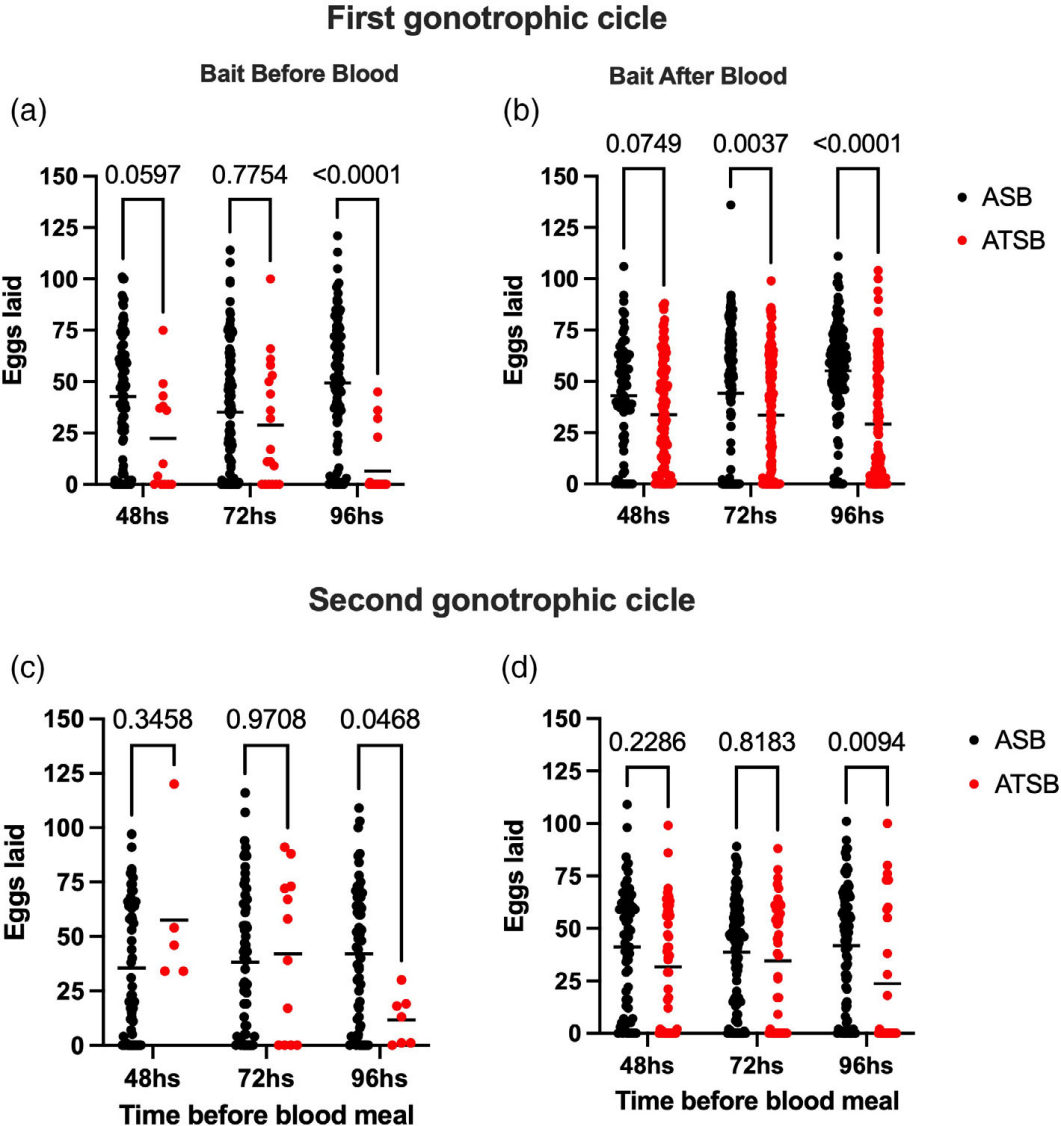
Number of eggs laid by *Aedes aegypti* females exposed to attractive toxic sugar baits (ATSBs) at different time points, that is, before (BB) and after (AB) blood feeding during the first cycle and only blood feeding during the second gonotrophic cycle to evaluate residual effects of previous treatments. Numbers over attractive sugar bait (ASB) and ATSB within a given interval indicate *p* values.

Overall, the number of eggs laid by females that ingested ATSB BB or AB was significantly reduced (54% and 32%, respectively) (*F* = 26.7, *p* < 0.0001; *F* = 53.05, *p* < 0.0001, respectively) during the first gonotrophic cycle (Figure 4a,b), particularly in the group that received ATSB 96 h BB and in the group that received ATSB 96 h AB.

During the second gonotrophic cycle, the number of eggs laid by females that had previously received ATSB 96 h AB remained lower compared with the ASB group (Figure 4c). The number of eggs laid by females that ingested ATSB 96 h AB followed a similar pattern, and overall, this group laid fewer eggs (29.9 eggs) compared with ASB‐fed females (40.5 eggs) (*F* = 10.6, *p* = 0.0012) (Figure 4d).

**FIGURE 5:**
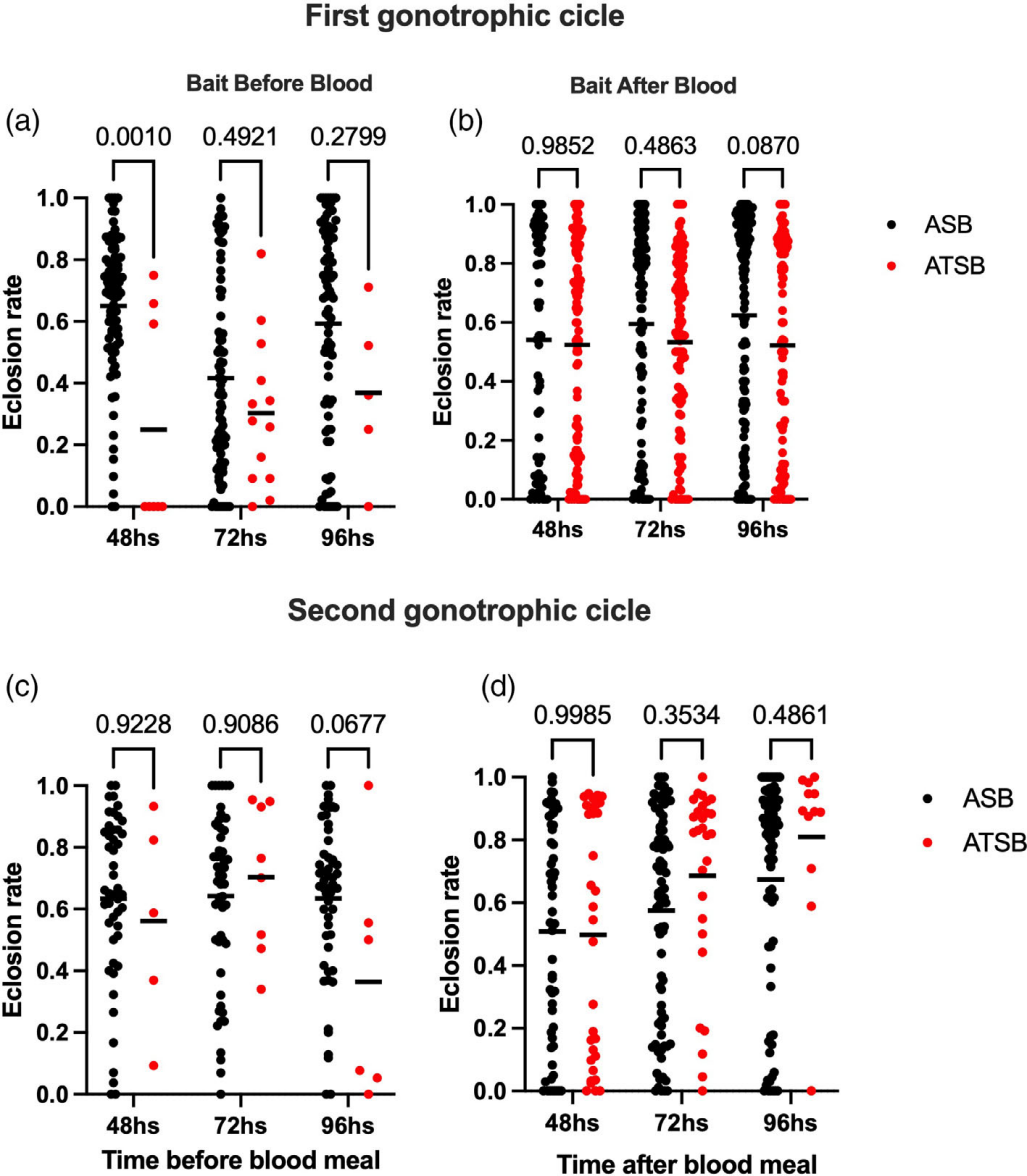
Eclosion rate from eggs laid by *Aedes aegypti* females exposed to attractive toxic sugar baits (ATSBs) at different time points, that is, before (BB) and after (AB) blood feeding during the first cycle and only blood feeding during the second gonotrophic cycle to evaluate residual effects of previous treatments. Numbers over attractive sugar bait (ASB) and ATSB within a given interval indicate *p* values.

The hatching rate of larvae from females that ingested ATSB BB or AB was significantly reduced (44% and 10%, respectively) (*F* = 13.99, *p* = 0.0002; *F* = 4.3, *p* = 0.04, respectively) compared with those that received ASB during the first gonotrophic cycle (Figure 5a,b).

In contrast, no significant differences in larval hatching were observed among experimental groups that received ATSB BB (*F* = 1.91, *p* = 0.17) (Figure 5c) or AB (*F* = 2.5, *p* = 0.11) (Figure 5d), when compared with ASB‐fed controls during the second gonotrophic cycle.

## DISCUSSION

ATSB‐IVM significantly reduced blood feeding of surviving females when ingested BB at 48–96 h, compared with females that received only ASB. In contrast, the ATSB‐IVM engorgement rate increased significantly 48 h AB, suggesting that females already engorged with blood were still able to feed on IVM‐containing baits. However, the effect of IVM on blood feeding did not persist beyond the first gonotrophic cycle. To our knowledge, no previous studies have examined the sublethal effects of IVM ingestion in sugar baits on blood feeding, as most ATSB‐IVM research has focused on determining lethal concentrations in *Ae. aegypti* (Tenywa et al., 2022) and *An. arabiensis* (Tenywa et al., 2017).

Overall, survival of females that ingested ATSB‐IVM either BB or AB was reduced across both gonotrophic cycles. The reduction, however, was much greater when bait was ingested BB, persisting until oviposition during the first gonotrophic cycle. The timing of ATSB‐IVM ingestion, specifically prior to blood feeding, may have suppressed protease activity, as reported in *Culex pipiens* Linnaeus, 1758 (Abdeltawab et al., 2020), thereby impairing blood digestion in *Ae. aegypti* females. The mosquitocidal effect of IVM is well established. In most studies, mosquitoes were exposed to IVM via blood meals (Deus et al., 2012; Focks et al., 1991; Hadlett et al., 2021; Whitehorn et al., 2013), whereas our approach—ingestion of ATSB‐IVM BB and AB—has not been previously reported. The concentration used in this study (15 ppm) was chosen to simulate partial ingestion of baits at higher doses, based on CL90 values reported by other authors, such as 260 ppm for *Ae. aegypti* (Dias et al., 2023), 300 ppm for *Ae. aegypti* (Tenywa et al., 2022) and 100 ppm for *An. arabiensis* (Tenywa et al., 2017), thus allowing us to observe sublethal effects on reproductive traits. Notably, the impact of ATSB‐IVM on survival was less pronounced in the second gonotrophic cycle. Similarly, Lyimo et al. (2017) reported that prior exposure to IVM‐containing blood reduced survival of *An. arabiensis* for up to 18 days after exposure, even following the first oviposition cycle.

Oviposition rate in *Ae. aegypti* was reduced when ATSB–IVM was ingested BB, compared with ASB‐fed controls. However, no effect was observed when ingestion occurred AB, indicating that the timing of exposure is critical. Although no previous studies have directly investigated the timing of IVM‐bait ingestion, ingestion of cattle blood containing IVM by *An. arabiensis* from an Ifakara (Tanzania) colony reduced oviposition by 60–80% compared with controls (Lyimo et al., 2017). Interestingly, this effect was not observed in a South African colony of the same species fed on cattle blood with similar IVM concentrations (Makhanthisa et al., 2021), suggesting differences in IVM susceptibility among mosquito populations (Kumar et al., 2023). Larger interspecific differences were reported by Dreyer et al. (2018), who showed that ingestion of IVM in mouse blood reduced oviposition only at higher concentrations (0.032 ppm in *An. stephensi* and 1.3 ppm in *Anopheles albimanus*). In the present study, oviposition rate was also reduced during the second gonotrophic cycle in females previously exposed to ATSB‐IVM in the first cycle, suggesting that IVM effects may persist across reproductive events.

Similar to oviposition, fecundity in *Ae. aegypti* was reduced when ATSB‐IVM was ingested either BB or AB. Although no studies have specifically addressed the effect of IVM‐containing sugar baits on fecundity, the use of ATSB‐IVM appears to mirror results from studies where IVM was administered through blood meals, albeit at much lower concentrations. For instance, Hadlett et al. (2021) reported a 65% reduction in egg production in *Ae. aegypti* fed on cattle blood containing only 0.5 ppm IVM, a concentration 30‐fold lower than in the present study. Similarly, Lyimo et al. (2017) and Makhanthisa et al. (2021) observed reductions of 63% and 75%, respectively, in *An. arabiensis* exposed to cattle blood containing 0.05 ppm IVM. Such reductions may result from impaired follicular development, leading to defective egg formation and incubation. Moreover, IVM has been shown to induce abnormally shaped eggs and increase the proportion of unhatched or sterile embryos, as reported by Mahmood et al. (1991) in *Ae. aegypti*.

ATSB‐IVM ingestion also reduced larval hatching; however, this effect appeared limited to intervals closer to blood feeding, particularly 48 h before the blood meal in the first gonotrophic cycle. An effect of IVM on egg viability may underlie this outcome; in Mahmood et al. (1991), *Ae. aegypti* larvae exhibited no apparent abnormalities but could not hatch. Moreover, the effects observed here resemble those of IVM ingestion through blood at much lower doses. For example, Hadlett et al. (2021) reported a 76% reduction in larval hatching in *Ae. aegypti* following ingestion of blood containing 0.5 ppm IVM.

The findings of this study have significant operational implications for *Ae. aegypti* control programs utilising ATSB‐IVM as a complementary tool. Beyond the direct mosquito mortality caused by IVM bait ingestion, this research demonstrates that a low concentration of 15 ppm is sufficient to cause substantial physiological disruption through sublethal effects. Specifically, the pronounced reduction in blood‐feeding rates (approximately 80%) and reproductive output (up to 80%) would likely lead to a significant decrease in population density when deployed in the field.

## CONCLUSION

This study demonstrates that ingestion of ATSBs containing IVM exerts significant sublethal effects on *Ae. aegypti*, reducing blood feeding, survival, oviposition, fecundity and larval hatching. The strongest impacts were observed when IVM was ingested BB and during the first gonotrophic cycle, although in some cases the effects persisted into the second reproductive cycle.

Whereas most previous studies with ATSB–IVM have focused on lethal endpoints, our findings expand this perspective by showing that sublethal concentrations can also disrupt key biological and reproductive parameters. These effects not only reduce direct survival but also decrease the likelihood of host–vector contact and compromise reproductive output.

## AUTHOR CONTRIBUTIONS

**Thais Alves de Moura:** Investigation; methodology; writing – original draft. **Alyne Cunha Alves Dias:** Investigation; methodology; writing – original draft. **Alexandre de Almeida e Silva:** Conceptualization; funding acquisition; writing – original draft; writing – review and editing.

## FUNDING INFORMATION

This study was supported by the Programa Pesquisa para o SUS: gestão compartilhada em saúde – PPSUS (Grant PPSUS FAPERO – 001/2020).

## CONFLICT OF INTEREST STATEMENT

The authors declare no conflict of interest.

## Data Availability

The data are available on Dryad website (Moura et al., 2025, https://doi.org/10.5061/dryad.q573n5txd).
